# Characterization of the complete chloroplast genome of ‘Quanhong poplar’ (*Populus deltoides* W. Bartram ex Humphry Marshall, 2011)

**DOI:** 10.1080/23802359.2024.2318391

**Published:** 2024-02-23

**Authors:** Weibing Zhuang, Yuhang Li, Xiaochun Shu, Zhong Wang, Yan Wang, Tao Wang

**Affiliations:** a Jiangsu Key Laboratory for the Research and Utilization of Plant Resources, Institute of Botany, Jiangsu Province and Chinese Academy of Sciences (Nanjing Botanical Garden Mem. Sun Yat-Sen), Nanjing, China; b Sishui Bureau of Natural Resources and Planning, Jining, China

**Keywords:** Chloroplast genome, phylogenetic analysis, Quanhong poplar, whole-genome sequencing

## Abstract

The color of the leaves is one of the most important factors for horticultural crops that are considered by breeders, and is also attracting more and more attention from economists and academics. 'Quanhong poplar’ (QHP), a rare, bright reddish-purple color-leaf cultivar that has been widely cultivated in China as a landscape tree, is a very precious color-leaf cultivar. In the present study, a reference-based assembly was performed using whole-genome sequencing data to characterize the chloroplast genome of 'QHP'. The total chloroplast genome size of ‘QHP’ is 156,950 bp, which is divided into two inverted repeat structures of 27,649 bp each, a small single-copy region of 16,563 bp, and a large single-copy region (LSC) of 85,089 bp. From the chloroplast genome, 130 genes have been predicted, including 85 protein-coding genes, 37 tRNA genes, and eight rRNA genes. A chloroplast genome containing 36.68% GC content was detected in 'QHP'. Three SNP sites have been developed between 'QHP' and *Populus deltoides* Zhonglin 2025. Based on the phylogenetic analysis of chloroplast genomes reported for *Populus*, the chloroplast of 'QHP' is closest to several strains of *Populus deltoides*.

## Introduction

The color-leaf plants are increasingly important in urban beautification, which also greatly contribute to societal and economic developments. The *Populus* plants, widely used in ecological shelterbelt, agricultural forest shelterbelt, and industrial timber forest, have the characteristics of fast growth, strong adaptability, easy reproduction, and cultivation. Combined with these benefits, color-leaf cultivars in *Populu*s have great potential for development in urban roads and landscaping, such as bright and reddish-purple leaves poplar (*Populus deltoides* cv. ‘Quanhong’), red-leaf poplar (*Populus deltoides* cv. ‘Zhonghong’), and bright red-leaf poplar (*Populus deltoides* cv. ‘Caihong’, *Populus deltoides* cv. ‘Zhongshancaiyun’). Due to its bright and attractive color and high ornamental value, ‘Quanhong poplar’ (QHP) is a treasure in the red-leaf tree species. Recently, the complete chloroplast genome sequence of *Populus deltoides* cv. ‘Caihong’ has been characterized (Zhuang et al. 2021). However, the complete chloroplast genome sequence of ‘QHP’ has not been evaluated. The present study characterizes the chloroplast genome sequence of ‘QHP’ in order to examine its physiology, molecular mechanisms, and phylogenetical relationships.

## Materials and methods

**Figure 1.**
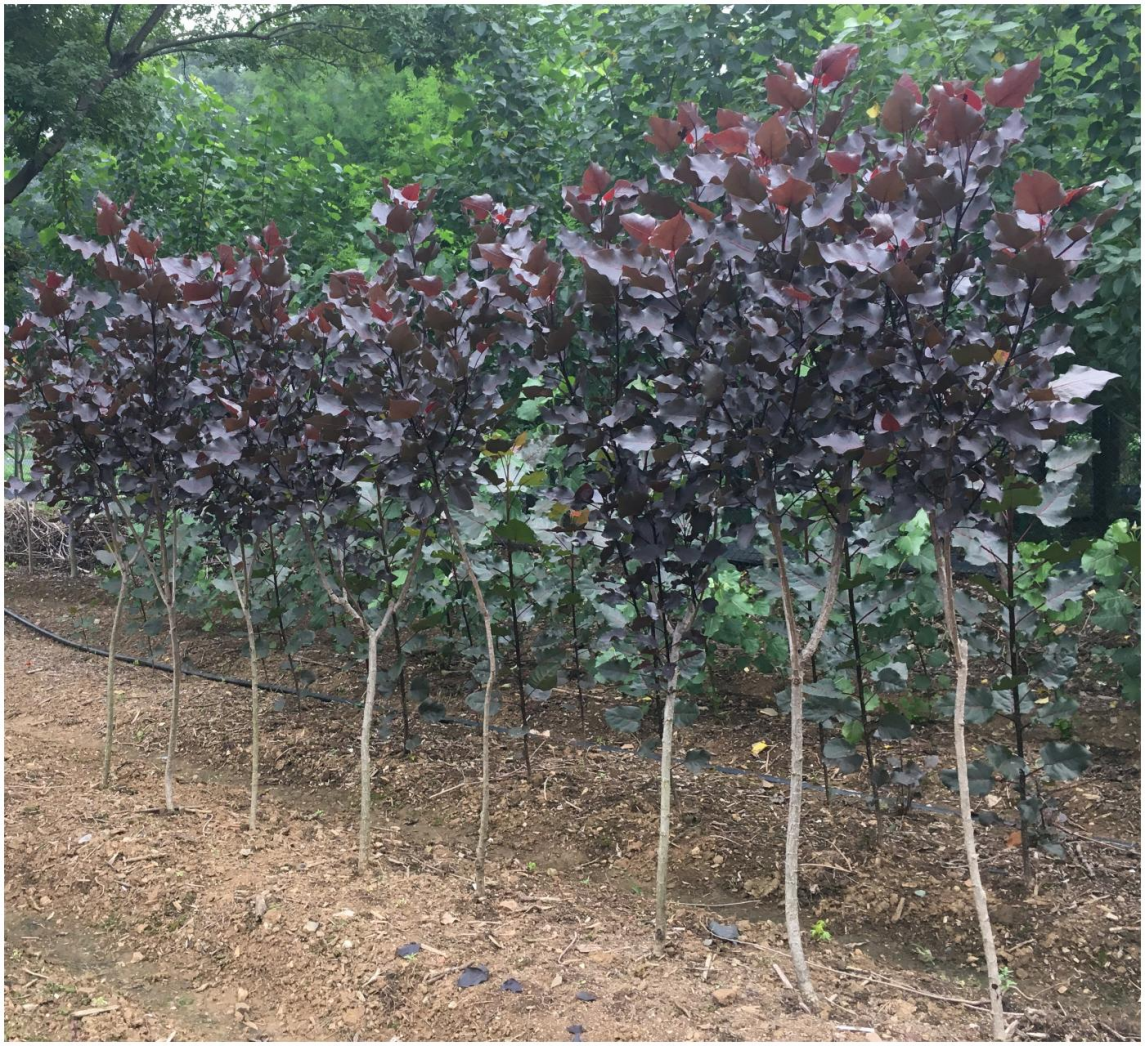
Morphology of QHP. The photo was taken by Weibing Zhuang at Nanjing Botanical Garden, Memorial Sun Yat-sen (E118_83, N32_06), Nanjing, China, on 24 June 2020, without any copyright issues. Leaves and new shoots of QHP were dark purple from bud flush in spring to late June, then showing a medium shade of purple from July to September, and turning into bright red in October. The bright and reddish-purple leaves among ‘QHP’ are the main characters distinct from other poplar cultivars.

The leaves of ‘QHP’ (Figure 1) were obtained from Nanjing Botanical Garden, Memorial Sun Yat-sen (E118_83, N32_06), Nanjing, China. The specimen of ‘QHP’ with voucher number NBG36 was deposited at the Herbarium of Nanjing Botanical Garden, Memorial Sun Yat-sen (http://www.jib.ac.cn/, Weibing Zhuang, weibingzhuangnj@sina.com). No endangered or protected species were involved in the study, and specific permissions were not required for the sample collection. Plant material collection followed local regulations and obtained local authorities’ approval. DNA was extracted using the plant DNA isolation reagent (Code: D9194, TaKaRa, Beijing, China) as per the provided instructions. After assessing DNA purity and integrity, qualified DNA underwent library construction and sequencing on the Illumina NovaSeq 6000 system (Illumina Inc., San Diego, CA). A total of 6136.1 Mb raw data were generated, with 3791.4 Mb of clean data utilized for chloroplast genome reconstruction. De novo genome assembly and annotation were performed using NOVOPlasty (Dierckxsens et al. 2017) and GeSeq (Tillich et al. 2017), respectively. The chloroplast genome sequence of *Populus trichocarpa* (NC_009143.1) served as a reference (Tuskan et al. 2006). The sequencing depth coverage was conducted by Geneious (Kearse et al. 2012). The map of chloroplast genome, cis-splicing genes and trans-splicing gene of 'QHP' was drawn by CPGview (http://www.1kmpg.cn/cpgview/, Liu et al. 2023). The SNP calling was performed using SNP-sites with the default options based on the multiple sequence alignments of chloroplast genome sequences (Page et al. 2016).

Multiple sequence alignments encompassing the complete chloroplast genomes of three poplar plants were executed using MAFFT to elucidate sequence disparities. The calculation of nucleotide variability (Pi) was conducted by DnaSP v6 with 600 bp window length and 200 bp step size (Rozas et al. 2017). In order to develop the novel molecular markers between 'QHP' and *Populus deltoides* Zhonglin 2025, distinctive primers were meticulously designed through Primer Premier 5.

A comprehensive phylogenetic analysis was undertaken using 23 complete chloroplast genomes from the '*Populus*’ genus, along with three Salix taxa included as outgroups. The sequences were aligned utilizing MAFFT v7.309 (Katoh and Standley 2013). For the purpose of model selection, the GTR-GAMMA (GTR + G) model was chosen through Modeltest (Posada and Crandall 1998), with the selection criterion being the Bayesian information content (BIC). The maximum-likelihood (ML) method with 1000 bootstraps was employed using MEGA-X software (Kumar et al. 2018).

## Results

**Figure 2.**
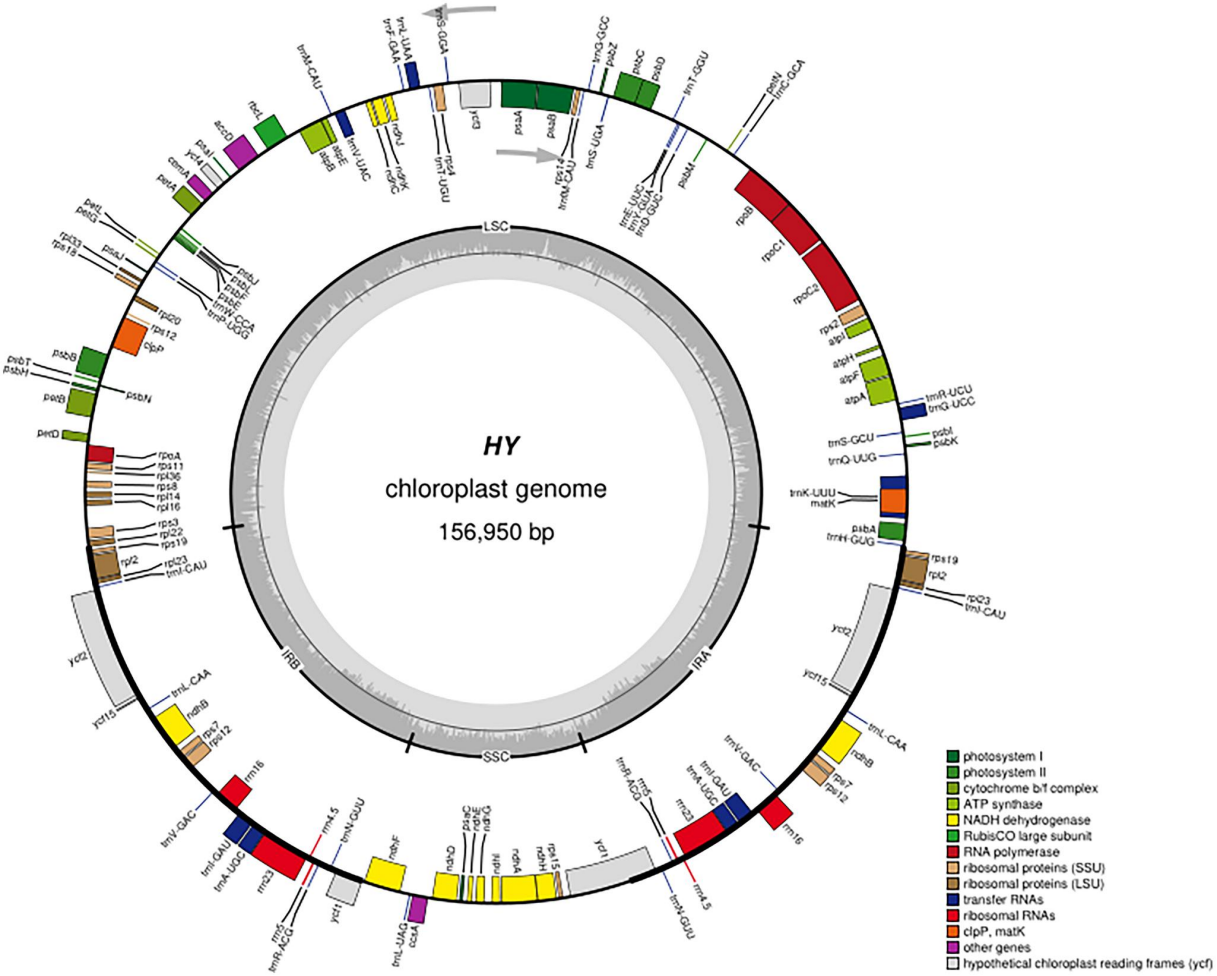
The chloroplast genome map of QHP. Genes shown outside the circle are transcribed clockwise, and genes inside are transcribed counter-clock-wise. Genes belonging to different functional groups are color-coded. The darker grey in the inner corresponds to the GC content and the lighter grey to the AT content.

**Figure 3.**
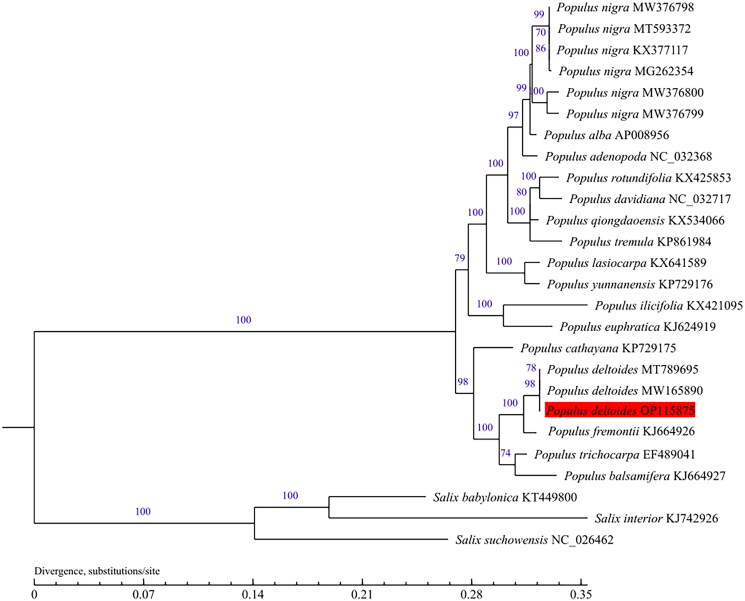
Phylogenetic tree was built with maximum-likelihood (ML) bootstrap analysis based on 23 chloroplast genome sequences from *Populus* and three taxa from Salix were served as outgroups. Label annotations against each species name represent the accession number on GenBank. The numbers above branches indicate bootstrap support values (%) of neighbor joining maximum likelihood trees, respectively. The bootstrap values are evaluated based on 1000 replications. The scale bar represents the number of substitutions at each locus. The following sequences were used: *Populus nigra* KX377117, *Populus cathayana* KP729175, *Populus tremula* KP861984, *Populus Alba* AP008956, *Populus yunnanensis* KP729176 (He et al. 2019), *Salix suchowensis* NC_026462, *Populus adenopoda* NC_032368 (Wu et al. 2020), *Populus balsamifera* KJ664927, *Populus euphratica* KJ624919, *Populus fremontii* KJ664926, *Populus ilicifolia* KX421095, *Populus lasiocarpa* KX641589, *Populus qiongdaoensis* KX534066, *Populus rotundifolia* KX425853, *Populus trichocarpa* EF489041 (Zhang et al. 2019), *Populus rotundifolia* KX425853, *Salix interior* KJ742926 (Zong et al. 2019), *Populus deltoides* MT789695, *Populus davidiana* NC_032717, *Salix babylonica* KT449800 (Zhuang et al. 2020), *Populus deltoides* MW165890 (Zhuang et al. 2021), *Populus nigra* MW376800, *Populus nigra* MW376799, *Populus nigra* MW376798 (Wang et al. 2022), and *Populus nigra* MG262354, *Populus nigra* MT593372 (unpublished).

The complete chloroplast genome of 'QHP' was assembled with a coverage of 3788× (Figure S1). The annotated chloroplast genome was submitted to GenBank under accession number OP115875. Raw sequencing reads were also deposited in the public repository SRA with accession number PRJNA 862560. The chloroplast genome size of 'QHP' measures 156,950 bp, comprising two inverted repeat regions (IRs) each spanning 27,649 bp, a large single-copy (LSC) region of 85,089 bp, and a small single-copy (SSC) region of 16,563 bp (Figure 2). The overall GC content of the 'QHP' chloroplast genome is 36.68%. Analysis revealed 130 genes predicted from this chloroplast genome, consisting of 85 protein-coding genes, 37 tRNA genes, and eight rRNA genes. In addition, the structures of the 12 protein-coding trans- and cis-splicing genes are shown in Figure S2. Comparative analysis of the complete chloroplast genome sequence of '*Populus deltoides*’ (accession number OP115875) was conducted against other cultivars, such as '*Populus fremontii*’ (KJ664926), '*Populus trichocarpa*’ (EF489041), and '*Populus deltoides*’ (MW165890), utilizing the online genome alignment tool mVISTA (Figure S3). The four genomic sequences exhibit notable similarity, yet substantial sequence variations were observed when comparing '*Populus deltoides*’ OP115875 with the other cultivars. These variations were primarily concentrated within the LSC and SSC regions (Figure S3). As is typical for most terrestrial plants, the IR regions demonstrated a higher degree of conservation than the LSC and SSC regions. As depicted in Figure 3, all *Populus* species formed a single large cluster, while the three distinct Salix varieties clustered together in a more compact arrangement. Notably, 'QHP' exhibited a close evolutionary kinship with several kinds of *Populus deltoides* (Figure 3).

In addition, sliding window analysis was conducted to reveal the highly variable regions in three *Populus deltoides* chloroplast genomes. The average value of nucleotide diversity (Pi) over the entire chloroplast genome was less than 0.01, indicating the whole cp genome was relatively conserved (Figure S4), which was consistent with the mVISTA result (Figure S3). The SNP calls based on the alignments of chloroplast genome sequences were evaluated (Excel S1). To develop the novel SNP sites between 'QHP' and *Populus deltoides* Zhonglin 2025, distinctive primers were designed in the regions with Pi > 0.001 (Table S1). The fragments were acquired with the expected size according to the agarose gel electrophoresis results (Figure S5). There was one base difference in the sequence of three different genes between 'QHP' and *Populus deltoides* Zhonglin 2025 (Table S1), which indicated that these three pairs of primers can be used as potential molecular markers.

## Discussion and conclusions

This is further underscored by the meticulous comparison of the 'QHP' plastome with preexisting published datasets, revealing a substantial degree of gene synteny shared with all publicly accessible data on different species of *Populus* spp. (Wang et al. 2016; Han et al. 2017; Zhuang et al. 2020, 2021). The comprehensive chloroplast genome sequence of 'QHP' holds significant value as a resource for DNA super-barcoding and the analysis of the phylogenetic origins within the genus '*Populus*’.

## Supplementary Material

Supplemental Material

Supplemental Material

## Data Availability

The genome sequence data that support the findings of this study are openly available in GenBank of NCBI at https://www.ncbi.nlm.nih.gov/ under the accession no. OP115875. The associated BioProject, SRA, and Bio-Sample numbers are PRJNA 862560, SRR16145711, and SAMN29984011, respectively.

## References

[CIT0001] Dierckxsens N, Mardulyn P, Smits G. 2017. NOVOPlasty: de novo assembly of organelle genomes from whole genome data. Nucleic Acids Res. 45(4):e18. doi:10.1093/nar/gkw955. 28204566 PMC5389512

[CIT0002] Han XM, Wang YM, Liu YJ. 2017. The complete chloroplast genome sequence of *Populus wilsonii* and its phylogenetic analysis. Mitochondrial DNA B Resour. 2(2):932–933. doi:10.1080/23802359.2017.1413291. 33474042 PMC7799498

[CIT0003] He Q, Zhang ZY, Wang WW. 2019. Characterization of the complete chloroplast genome of black poplar (*Populus nigra* L.). Mitochondrial DNA B. 4(1):1261–1262. doi:10.1080/23802359.2019.1591241.

[CIT0004] Katoh K, Standley DM. 2013. MAFFT multiple sequence alignment software version 7: improvements in performance and usability. Mol Biol Evol. 30(4):772–780. doi:10.1093/molbev/mst010. 23329690 PMC3603318

[CIT0005] Kearse M, Moir R, Wilson A, Stones-Havas S, Cheung M, Sturrock S, Buxton S, Cooper A, Markowitz S, Duran C, et al. 2012. Geneious Basic: an integrated and extendable desktop software platform for the organization and analysis of sequence data. Bioinformatics. 28(12):1647–1649. doi:10.1093/bioinformatics/bts199. 22543367 PMC3371832

[CIT0006] Kumar S, Stecher G, Li M, Knyaz C, Tamura K. 2018. MEGA X: molecular evolutionary genetics analysis across computing platforms. Mol Biol Evol. 35(6):1547–1549. doi:10.1093/molbev/msy096. 29722887 PMC5967553

[CIT0007] Liu S, Ni Y, Li J, Zhang X, Yang H, Chen H, Liu C. 2023. CPGView: a package for visualizing detailed chloroplast genome structures. Mol Ecol Resour. 23(3):694–704. doi:10.1111/1755-0998.13729. 36587992

[CIT0008] Posada D, Crandall KA. 1998. MODELTEST: testing the model of DNA substitution. Bioinformatics. 14(9):817–818. doi:10.1093/bioinformatics/14.9.817. 9918953

[CIT0009] Page AJ, Taylor B, Delaney AJ, Soares J, Seemann T, Keane JA, Harris SR. 2016. SNP-sites: rapid efficient extraction of SNPs from multi-FASTA alignments. Microb Genom. 2(4):000056. 10.1099/mgen.0.000056 PMC5320690 28348851

[CIT0010] Rozas J, Ferrer-Mata A, Sánchez-DelBarrio JC, Guirao-Rico S, Librado P, Ramos-Onsins SE, Sánchez-Gracia A. 2017. DnaSP 6: DNA sequence polymorphism analysis of large data sets. Mol Biol Evol. 34(12):3299–3302. doi:10.1093/molbev/msx248. 29029172

[CIT0011] Tillich M, Lehwark P, Pellizzer T, Ulbricht-Jones ES, Fischer A, Bock R, Greiner S. 2017. GeSeq - versatile and accurate annotation of organelle genomes. Nucleic Acids Res. 45(W1):W6–W11. doi:10.1093/nar/gkx391. 28486635 PMC5570176

[CIT0012] Tuskan GA, Difazio S, Jansson S, Bohlmann J, Grigoriev I, Hellsten U, Putnam N, Ralph S, Rombauts S, Salamov A, et al. 2006. The genome of black cottonwood, *Populus trichocarpa* (Torr. & Gray). Science. 313(5793):1596–1604. doi:10.1126/science.1128691. 16973872

[CIT0013] Wang TJ, Fan LQ, Guo XY, Luo X, Wang K. 2016. Characterization of the complete chloroplast genome of *Populus qiongdaoensis* T. Hong et P. Luo. Conserv Genet Resour. 8(4):435–437. doi:10.1007/s12686-016-0590-3.

[CIT0014] Wang Y, Huang J, Li E, Xu S, Zhan Z, Zhang X, Yang Z, Guo F, Liu K, Liu D, et al. 2022. Phylogenomics and biogeography of *Populus* based on comprehensive sampling reveal deep-level relationships and multiple intercontinental dispersals. Front Plant Sci. 13:813177. doi:10.3389/fpls.2022.813177. 35185985 PMC8855119

[CIT0015] Wu DY, Wang YP, Wu DH, Dou LJ, Gao LM. 2020. Single-molecule sequencing yields the complete chloroplast genome sequence of *Populus deltoides*. Mitochondrial DNA B Resour. 5(1):125–126. doi:10.1080/23802359.2019.1698346. PMC7720975 33366451

[CIT0016] Zhang Y, Zong D, Zhou AP, He CZ. 2019. The complete chloroplast genome of *Populus tomentosa* narrow crown, native to Northern China. Mitochondrial DNA B. 4(1):1208–1210. doi:10.1080/23802359.2018.1553531.

[CIT0017] Zhuang W, Shu X, Zhang M, Wang T, Zhang F, Wang N, Wang Z. 2020. Characterization of the complete chloroplast genome of *Populus deltoides* Zhonglin 2025. Mitochondrial DNA B. 5(3):3705–3706. doi:10.1080/23802359.2020.1833773. PMC7671709 33367076

[CIT0018] Zhuang WB, Shu XC, Zhang H, Wang T, Zhang FJ, Wang N, Wang Z. 2021. Complete chloroplast genome sequence and phylogenetic analysis of *Populus deltoides* Caihong. Mitochondrial DNA B Resour. 6(2):389–390. doi:10.1080/23802359.2020.1869612. 33659687 PMC7872566

[CIT0019] Zong D, Zhou AP, Li D, He CZ. 2019. The complete chloroplast genome of *Populus xiangchengensis*, an endemic species in Southwest China. Mitochondrial DNA B. 4(1):70–71. doi:10.1080/23802359.2018.1536463.

