# Supplementary material for: Characterization of the complete chloroplast genome of ‘Quanhong poplar’ (Populus deltoides W. Bartram ex Humphry Marshall, 2011)
Source: Mitochondrial DNA B Resour. 2024 Feb 23;9(2):285–9. doi: 10.1080/23802359.2024.2318391 (PMC10896124; doi:10.1080/23802359.2024.2318391)
Supplement: Supplemental Material [file TMDN_A_2318391_SM9335.docx]

**Supplementary material**

**Figure S1.** The sequencing depth of coverage plots of QHP.


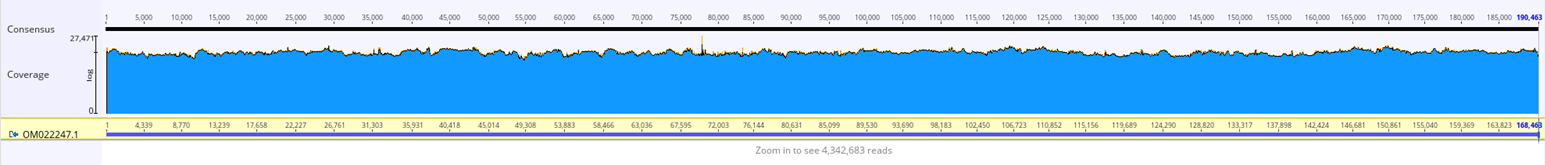


**Figure S2.** Schematic map of the 11 cis-splicing genes(A) and one trans splicing gene(B) in the chloroplast genome of QHP. The exons are shown in black; the introns are shown in white. The arrow indicates the sense direction of the gene. The map was generated using CPGview

(A)


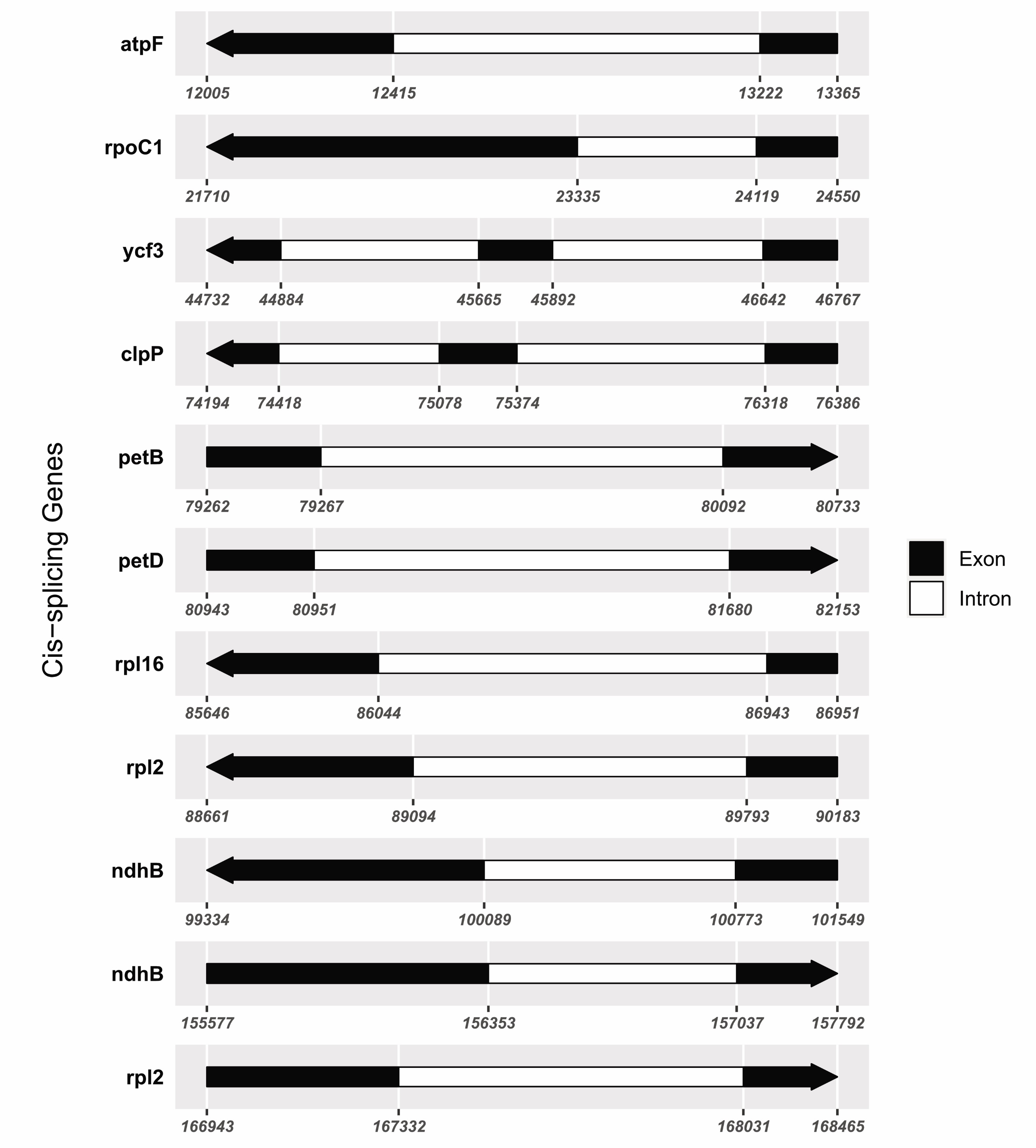


(B)


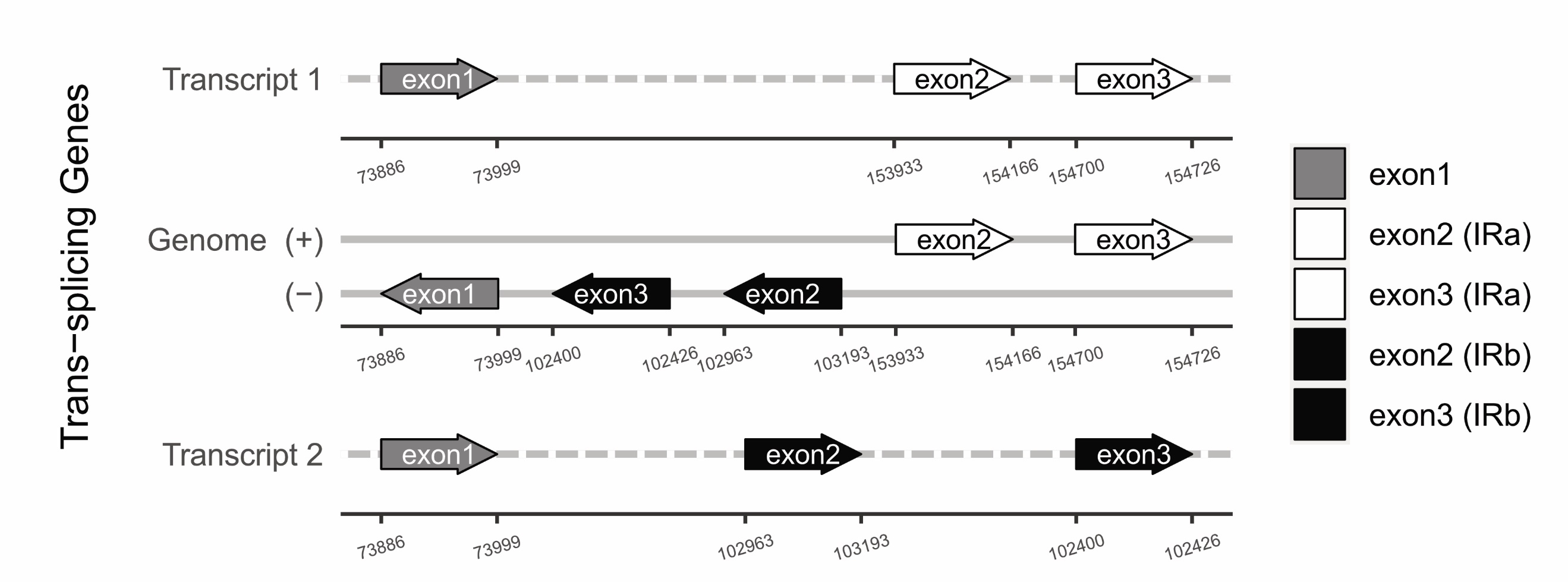


**Figure S3.** Comparison of four chloroplast genomes using the mVista alignment program, with *Populus trichocarpa* (EF489041) as a reference. The x-axis means the midpoint of the window, and the y-axis means nucleotide diversity. Genome regions are color-coded as protein-coding, rRNA coding, tRNA coding, or conserved noncoding sequences.


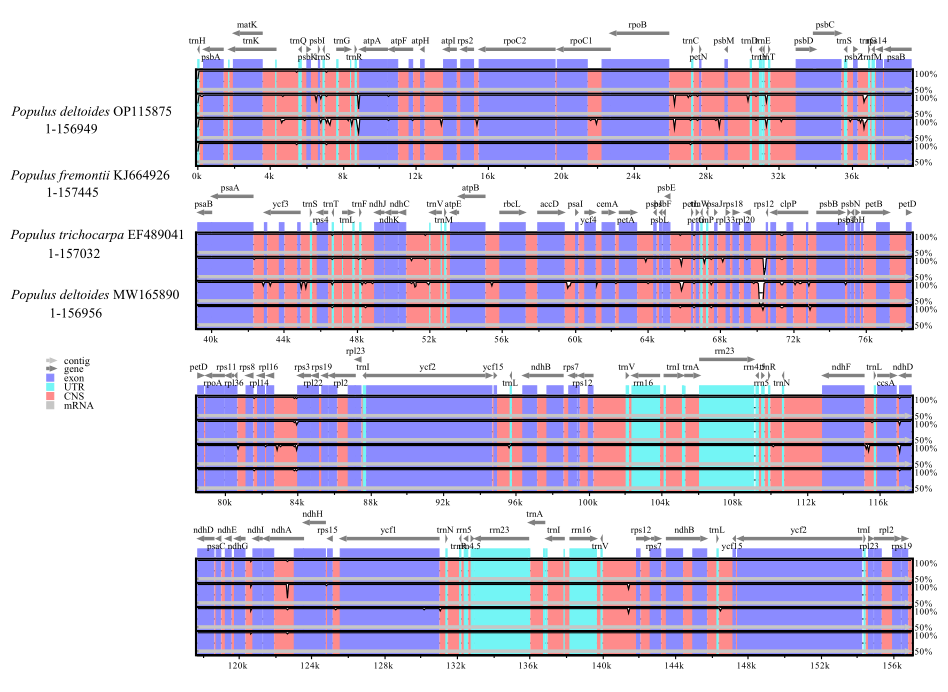


**Figure S4.** Nucleotide diversity (Pi) among cp genomes of three *Populus deltoides* species. The X-axis represents the position of the midpoint of a window, while the Y-axis represents nucleotide variability (Pi) of each window (window length: 600 bp, step size: 200 bp).


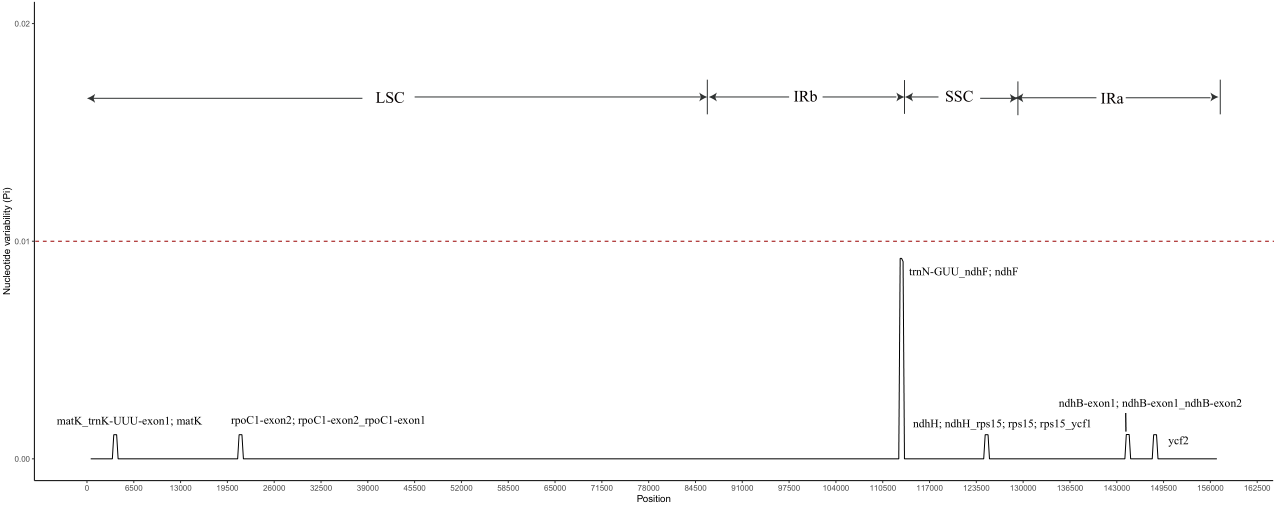


**Figure S5.** Three pairs of primers were detected by agarose gel electrophoresis.1, 3, 5: fragments obtained from leaves of 'QHP' with the primers marker 1, marker 2 and marker 3, respectively; 2, 4, 6: fragments obtained from leaves of *Populus deltoides*Zhonglin 2025 with the primers marker 1, marker 2 and marker 3, respectively.


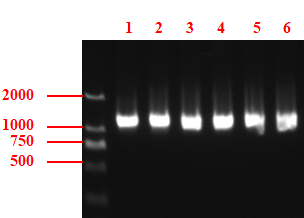


**Table S1.** PCR primers designed according to the mutational hotspots and corresponding sequenced products within two *Populus* *deltoides* species.

| Mutational hotspots | PCR primers | Product |
| --- | --- | --- |
| 'QHP':3001-4400 | TCTAGATGGATGGGGTACGGT | TCTAGATGGATGGGGTACGGTATTAGTCCCTCTGACAGATAATTTAAATGTGGGAATTTGCCCTCTAAAAAAGGAAATATTGAATGAATTGATCGTAAATTATGAGATTTTACTATTTCTGATCTTTCTAAAGAGGATACTAATCGTAAGGAAAATGGAATTTCCACAATAACTGCAAACCCCTCCGATATCATTTGAAAATACAAATTTTTGTTATACCTAAAAAATGTATTTTGGTTAGAATCATTAGCAGAAATAATCAAATGATTCTGTTGATACATTCGAGTAATTAAACGTTTTACGATTAGTAAACTATATTTATTGTCATAACCTACATTTTCTAACAAAATAGATCTATTTAAGTCACGATCATGAGCAAATGTATAAATATACTCCCGAAAGATAAGTGGGTATAGGAAGTCATTTTTTCGAGATCTATCTATTTCTAAATTTCTTTGAGATTTCTCTATTTTCATTTTTAATTCGATTTGATTGAAGCAAAGATAGGGAGTTTATTGGGTTATTAAATGATACATAGTGCGATACCATAAAAACAAAATAGTATAATATAAAAAGAATGGATACCTCGGAAATAGTTAAACTCACCAACGGA**T**CCTCTATCCTCTCTTTTTTCCATCTAATTGGTTTATGTTCATTTCTAATTGGTTTATGTTCATTTATAGGGTAATAGGTGACTAGAAATCCTTTACTTTTTCAAGTCAATCACTCTTTTTTGATTTTGGAAAAAAAAAATTGCTTTATCAATATACTTTTTCTTCTACACATTCAACTCCCCTTCATAGTGGAGAATAGCTAATAGTTAGGACTCATTAAAAAAATCGAAAATCCACTCAAGAAAAAGCTTTTCCCGCACTAGGCACTAATCCATTTTTAACGTCTAATTAGATCGGAAAATCATTCAAATTAAGAACGGGAGCTCGTTGCTTTTTTATTTCCCTATAATTGGAGCCGCGGAGCTCTGTCCATTTATTAACTCGACCCAACCCCAACTTTGAATTTGAATTCATTTGTTTTTATGTTACGCACCAAGAATTCAAACAAGGTTTGTTTGGAGCCGATCCGGTAAGAATCAAATATTCTCAGAATTCTCCATTGATACGACATGCTGTTTTTTCCATTCATTCCTTTCAGGATCAGTCGTGGTCTTACAAATAATACCGATGGTATGGACGAATCCC |
|  | GGGATTCGTCCATACCATCGG |  |
| *Populus deltoids* Zhonglin 2025: 3000-4399 | TCTAGATGGATGGGGTACGGT | TCTAGATGGATGGGGTACGGTATTAGTCCCTCTGACAGATAATTTAAATGTGGGAATTTGCCCTCTAAAAAAGGAAATATTGAATGAATTGATCGTAAATTATGAGATTTTACTATTTCTGATCTTTCTAAAGAGGATACTAATCGTAAGGAAAATGGAATTTCCACAATAACTGCAAACCCCTCCGATATCATTTGAAAATACAAATTTTTGTTATACCTAAAAAATGTATTTTGGTTAGAATCATTAGCAGAAATAATCAAATGATTCTGTTGATACATTCGAGTAATTAAACGTTTTACGATTAGTAAACTATATTTATTGTCATAACCTACATTTTCTAACAAAATAGATCTATTTAAGTCACGATCATGAGCAAATGTATAAATATACTCCCGAAAGATAAGTGGGTATAGGAAGTCATTTTTTCGAGATCTATCTATTTCTAAATTTCTTTGAGATTTCTCTATTTTCATTTTTAATTCGATTTGATTGAAGCAAAGATAGGGAGTTTATTGGGTTATTAAATGATACATAGTGCGATACCATAAAAACAAAATAGTATAATATAAAAAGAATGGATACCTCGGAAATAGTTAAACTCACCAACGGA**C**CCTCTATCCTCTCTTTTTTCCATCTAATTGGTTTATGTTCATTTCTAATTGGTTTATGTTCATTTATAGGGTAATAGGTGACTAGAAATCCTTTACTTTTTCAAGTCAATCACTCTTTTTTGATTTTGGAAAAAAAAAATTGCTTTATCAATATACTTTTTCTTCTACACATTCAACTCCCCTTCATAGTGGAGAATAGCTAATAGTTAGGACTCATTAAAAAAATCGAAAATCCACTCAAGAAAAAGCTTTTCCCGCACTAGGCACTAATCCATTTTTAACGTCTAATTAGATCGGAAAATCATTCAAATTAAGAACGGGAGCTCGTTGCTTTTTTATTTCCCTATAATTGGAGCCGCGGAGCTCTGTCCATTTATTAACTCGACCCAACCCCAACTTTGAATTTGAATTCATTTGTTTTTATGTTACGCACCAAGAATTCAAACAAGGTTTGTTTGGAGCCGATCCGGTAAGAATCAAATATTCTCAGAATTCTCCATTGATACGACATGCTGTTTTTTCCATTCATTCCTTTCAGGATCAGTCGTGGTCTTACAAATAATACCGATGGTATGGACGAATCCC |
|  | GGGATTCGTCCATACCATCGG |  |
| 'QHP': 20401-21800 | CCCCGCATAACCTCCTGAAG | CCCCGCATAACCTCCTGAAGTATTCCCCATACAATTGGCTCTTTTTCTCGAATTTTACTCTTAGCAACTCCTATGTTTGAAGCAAGATGTTGCCTAATTAGACCACGAATTACAAATGTCTGGAAAAGCTCTATTGCTATTTCGCGAGGCAATCCACATCGATGTAATGAAAGTGAGGGTCCTACGACAATGACAGAACGCCCCGAATAATCAACCCGTTTGCCAAGCATAGTCTCACGAAACCTTCCCTCTTTGCCTTCAATTACATCTGAAAACGACTTGTAAACCTTATTATGACCGTCTCTCATTGGTTGTCCGCGGATTCCATTATCAAGAAGTGTATCTACGGCTTCTTGTACCAATTTCTCCTGACACATTACTAATTCCCCTGGCGTAGATCTACTTGTTGTTAATAGATCCGTAAGAGTATTGTTCCGATAAATAACTCTTCTATAGAGTTCATTAATATCTGAACTCATTAGTTTACCTCCATCTATCTGAATGATGGGTCTCAATTCAGGAGGAAGAACTGGTAATAGACATAAAACCATCCATTCTGGTTCTATATTTGTTCGAATAAAATGCTTAGCTAATTCCACGCGTCTAACTAAAAAATCCTTTCTTCTTCCAACTTTTCGATCTTCCCAT**G**CATTCCCGGTGGGTCCTTCTTCCCCTAATTCTTTCCATTCTAACGACGAATAATCTAGAATAATTCGCAAATCCAGATCGGCTAATAGTTCTCGGATAGCACCAGCTCCAGTAGAAATTTCTCGATTTCGAAATGTATCGAACCCTTGGGTAGTAAAAAAAAGCGGAATACTGTATTTCCAAGATTGTATTTCATATTCGAACGAACCTCGTAATCGTAAGAAAGTAGGTTTTTTGGCTATGGGCCTAGCAAAAGAAAAATTGGGATAGGATCCTATAGGATCTCCCCCCTTCAAAATCGGACGTGAAAGTTTCCTTTCATCCGGCTCAAGTAGTTACACCAAATAAAGATAAAATAAAGGGTTCCTGCTTTCAAAATGGATAAAACCCCCCAAAAAAAAGGAGCCACTCCTTACTCAAGTTCCCAATGAAGACCAAGCAACATTTCATTGATTCATTCTTCTTTTCTGTTTTTTTTTTTTTAGAATTTCTAAATTCTTTATTCAATTATCACAATTACGACCTAAATGCAATGCGA |
|  | TCGCATTGCATTTAGGTCGT |  |
| *Populus deltoids* Zhonglin 2025: 20400-21799 | CCCCGCATAACCTCCTGAAG | CCCCGCATAACCTCCTGAAGTATTCCCCATACAATTGGCTCTTTTTCTCGAATTTTACTCTTAGCAACTCCTATGTTTGAAGCAAGATGTTGCCTAATTAGACCACGAATTACAAATGTCTGGAAAAGCTCTATTGCTATTTCGCGAGGCAATCCACATCGATGTAATGAAAGTGAGGGTCCTACGACAATGACAGAACGCCCCGAATAATCAACCCGTTTGCCAAGCATAGTCTCACGAAACCTTCCCTCTTTGCCTTCAATTACATCTGAAAACGACTTGTAAACCTTATTATGACCGTCTCTCATTGGTTGTCCGCGGATTCCATTATCAAGAAGTGTATCTACGGCTTCTTGTACCAATTTCTCCTGACACATTACTAATTCCCCTGGCGTAGATCTACTTGTTGTTAATAGATCCGTAAGAGTATTGTTCCGATAAATAACTCTTCTATAGAGTTCATTAATATCTGAACTCATTAGTTTACCTCCATCTATCTGAATGATGGGTCTCAATTCAGGAGGAAGAACTGGTAATAGACATAAAACCATCCATTCTGGTTCTATATTTGTTCGAATAAAATGCTTAGCTAATTCCACGCGTCTAACTAAAAAATCCTTTCTTCTTCCAACTTTTCGATCTTCCCAT**T**CATTCCCGGTGGGTCCTTCTTCCCCTAATTCTTTCCATTCTAACGACGAATAATCTAGAATAATTCGCAAATCCAGATCGGCTAATAGTTCTCGGATAGCACCAGCTCCAGTAGAAATTTCTCGATTTCGAAATGTATCGAACCCTTGGGTAGTAAAAAAAAGCGGAATACTGTATTTCCAAGATTGTATTTCATATTCGAACGAACCTCGTAATCGTAAGAAAGTAGGTTTTTTGGCTATGGGCCTAGCAAAAGAAAAATTGGGATAGGATCCTATAGGATCTCCCCCCTTCAAAATCGGACGTGAAAGTTTCCTTTCATCCGGCTCAAGTAGTTACACCAAATAAAGATAAAATAAAGGGTTCCTGCTTTCAAAATGGATAAAACCCCCCAAAAAAAAGGAGCCACTCCTTACTCAAGTTCCCAATGAAGACCAAGCAACATTTCATTGATTCATTCTTCTTTTCTGTTTTTTTTTTTTTAGAATTTCTAAATTCTTTATTCAATTATCACAATTACGACCTAAATGCAATGCGA |
|  | TCGCATTGCATTTAGGTCGT |  |
| 'QHP': 123993-125392 | GTACTCCGGAAGCTCGTAGC | GTACTCCGGAAGCTCGTAGCATTGGTCCTGATAAACCCCAATTTATTGCTTCCTCTGCACTAACAATACCTATTCCTTCAACGCGTTCTAAAAAAATAGGATTTCGCGTAATAAGTTTTTGATATTCAGCAACTCCTGTTAAAAAATAATCGCAGAAATCCAAACATTTATCTAGCCAGCCATGAGGTAGATCAGCTGCTACTCCTCCGATACGAAAATAATTATGCATCATTCTCATACCAGTGGCAGCTTCGAATAAATCATATATTAACTCTCTTTCTCTAAAAATATAGAAGAAAGGGGTCTGCCCACCAATATCTGCCATAAAAGGGCCAAGCCATAAGAGATGAGAAGCTATACGACTCAACTCCAACATAATTACTCTGATATAGCTAGCTCTTTTAGGTACTTGAATATTTCCCAACTGTTCCGGTCCATTTATTGTTATCGCTTCTGTAAACATAGTAGCTAAATAATCCCAACGTGTTACATAAGGCAAATATTGTATAATTGTTCGGTTTTCCGCAATTTTTTCCATCCCTCTGTGTAAATAACCTAATATTGGTTCGCAGTCAATAACATCTTCACCGTCTAGACTAAGGATGAGTCGAAGAACGCCATGCATTGATGGGTGGTGGGGACCCATATTGACTATCATAAAGTCCTTTCTTGTAGCTGGTACATTCATAGGGGGTTCCTCGATTT**A**TTTTTCCATGAATTACTGAAAACGAAAAGAAGTTCATCAAAATTCAAGTTTAAGATCTAATAAATCAAATAATAAAAAAAAAGACTCTTCAAATTAACGAGTTTTTGATTCCCGAATGTTCAACTGGCTAATTAATTCTTTATAACGTACTCCATTTTTCTTTGCCAAATAAGACAGTAGTCGTTGGCGTTTTCCTAGAATTTTCCGTAAACCTCTTTGAGATAAATAGTCTTTTCTATGTAATTCCAAATGTGAAGTAAGTCTTCGTATCTTATTAGTAAAACTTACTATTTGAAATTCAACGGATCCCTTGTTTTCTTTTTTGTCTTCTTGTGAAATAATTGAAATGAATGAACTTTTTACCATAAAATGAAATCCCCCTCCTCCCGCCTTTTTAAGATAGTTTTATTGATCAGTAATAATAAATAATGGAATATTAAATAAGAATGTCAGTTTGTTTGAATTTGGT |
|  | ACCAAATTCAAACAAACTGACA |  |
| *Populus deltoids* Zhonglin 2025: 124000-125399 | GTACTCCGGAAGCTCGTAGC | GTACTCCGGAAGCTCGTAGCATTGGTCCTGATAAACCCCAATTTATTGCTTCCTCTGCACTAACAATACCTATTCCTTCAACGCGTTCTAAAAAAATAGGATTTCGCGTAATAAGTTTTTGATATTCAGCAACTCCTGTTAAAAAATAATCGCAGAAATCCAAACATTTATCTAGCCAGCCATGAGGTAGATCAGCTGCTACTCCTCCGATACGAAAATAATTATGCATCATTCTCATACCAGTGGCAGCTTCGAATAAATCATATATTAACTCTCTTTCTCTAAAAATATAGAAGAAAGGGGTCTGCCCACCAATATCTGCCATAAAAGGGCCAAGCCATAAGAGATGAGAAGCTATACGACTCAACTCCAACATAATTACTCTGATATAGCTAGCTCTTTTAGGTACTTGAATATTTCCCAACTGTTCCGGTCCATTTATTGTTATCGCTTCTGTAAACATAGTAGCTAAATAATCCCAACGTGTTACATAAGGCAAATATTGTATAATTGTTCGGTTTTCCGCAATTTTTTCCATCCCTCTGTGTAAATAACCTAATATTGGTTCGCAGTCAATAACATCTTCACCGTCTAGACTAAGGATGAGTCGAAGAACGCCATGCATTGATGGGTGGTGGGGACCCATATTGACTATCATAAAGTCCTTTCTTGTAGCTGGTACATTCATAGGGGGTTCCTCGATTT**C**TTTTTCCATGAATTACTGAAAACGAAAAGAAGTTCATCAAAATTCAAGTTTAAGATCTAATAAATCAAATAATAAAAAAAAAGACTCTTCAAATTAACGAGTTTTTGATTCCCGAATGTTCAACTGGCTAATTAATTCTTTATAACGTACTCCATTTTTCTTTGCCAAATAAGACAGTAGTCGTTGGCGTTTTCCTAGAATTTTCCGTAAACCTCTTTGAGATAAATAGTCTTTTCTATGTAATTCCAAATGTGAAGTAAGTCTTCGTATCTTATTAGTAAAACTTACTATTTGAAATTCAACGGATCCCTTGTTTTCTTTTTTGTCTTCTTGTGAAATAATTGAAATGAATGAACTTTTTACCATAAAATGAAATCCCCCTCCTCCCGCCTTTTTAAGATAGTTTTATTGATCAGTAATAATAAATAATGGAATATTAAATAAGAATGTCAGTTTGTTTGAATTTGGT |
|  | ACCAAATTCAAACAAACTGACA |  |
